# Laser-induced metastable mixed phase of AuNi nanoparticles: a coherent X-ray diffraction imaging study

**DOI:** 10.1107/S1600577520001617

**Published:** 2020-03-31

**Authors:** Yoonhee Kim, Chan Kim, Kangwoo Ahn, Jungwon Choi, Su Yong Lee, Hyon Chol Kang, Do Young Noh

**Affiliations:** a European X-ray Free Electron Laser Facility, Holzkoppel 4, 22869 Schenefeld, Germany; b Department of Physics and Photon Science and School of Materials Science and Engineering, Gwangju Institute of Science and Technology, 61005 Gwangju, Korea; c Pohang Accelerator Laboratory, 37673 Pohang, Korea; d Department of Materials Science and Engineering, Chosun University, 61452 Gwangju, Korea

**Keywords:** AuNi nanocrystals, metastable mixed phase, CXDI, laser irradiation

## Abstract

The laser annealing process for AuNi nanoparticles has been visualized using coherent X-ray diffraction imaging (CXDI). AuNi bimetallic alloy nanoparticles, originally phase separated due to the miscibility gap, transform to metastable mixed alloy particles with rounded surface as they are irradiated by laser pulses. A three-dimensional CXDI shows that the internal part of the AuNi particles is in the mixed phase with preferred compositions at ∼29 at% of Au and ∼90 at% of Au.

## Abstract 2

Coherent diffraction imaging in the forward-scattering geometry reveals AuNi nano alloy particles in a metastable mixed phase. The AuNi nanoparticles irradiated by nanosecond laser pulses exhibit the mixed phase overcoming the miscibility gap.

## 1. Introduction   

Over the last few decades, intermetallic alloy systems have attracted great attention due to their remarkable physicochemical properties and applications (Lee *et al.*, 2015 ▸; Kang *et al.*, 2013 ▸; Stamenkovic *et al.*, 2007 ▸). Nickel-based bimetallic alloys are so far the most commonly used catalysts in steam reforming processes. Compared with Ni, Ni bimetallic alloys exhibit superior catalytic performances, *e.g.* high activity, enhanced lifetime, high resistance to carbon formation, and highly selective H_2_ conversion (Besenbacher *et al.*, 1998 ▸; Wu *et al.*, 2013 ▸). The physicochemical properties of alloy systems are strongly affected by their composition, crystal shape, domain structure, and phase boundary. Recent studies on the effects of domain structure, *e.g.* nano-twinning in alloys (Li *et al.*, 2018 ▸), ultra-fine nano-grain structure (Hu *et al.*, 2017 ▸), and dual-phase nano-structuring (Wu *et al.*, 2017 ▸), proved that controlling the domain structure is crucial in improving metallic properties.

One of the distinguishing features of AuNi alloy systems is the miscibility gap that exists throughout the whole composition range of elements near the phase transition temperature (Reichert *et al.*, 2005 ▸; Sopoušek *et al.*, 2017 ▸). As a result, in equilibrium, AuNi nanoparticles exist in a phase-separated state in which Au-rich and Ni-rich regions are separated. To overcome the miscibility gap, pulsed laser annealing processes have recently been introduced (Swiatkowska-Warkocka *et al.*, 2015 ▸; Pyatenko *et al.*, 2013 ▸) which can be used to synthesize new metastable nano-alloys. The averaged atomic composition and domain structural information are readily accessible by typical X-ray diffraction techniques. Surface-sensitive structural information is observable with electron microscopies which have intrinsic limitations due to the short penetration depth of electrons (Stamenkovic *et al.*, 2007 ▸; Besenbacher *et al.*, 1998 ▸; Li *et al.*, 2018 ▸; Hu *et al.*, 2017 ▸; Wu *et al.*, 2017 ▸; Kim *et al.*, 2008 ▸). A more detailed understanding of laser-induced structural dynamics is still in demand.

Here, we present a visualization of the phase boundaries inside the particles and the rounding effect of crystal facets of AuNi nanoparticles under laser irradiation by using coherent X-ray diffraction imaging (CXDI) (Ayyer *et al.*, 2016 ▸; Ulvestad *et al.*, 2015 ▸; Lo *et al.*, 2018 ▸; Mastropietro *et al.*, 2017 ▸; Donnelly *et al.*, 2017 ▸; Kim *et al.*, 2018 ▸). The pulsed laser annealing transforms phase-separated AuNi nanoparticles to metastable mixed AuNi alloy nanoparticles. The crystal shape of AuNi alloy crystals becomes a non-equilibrium spherical shape as the number of the irradiated laser pulses increase.

## 2. Experiment setup and sample preparation   

**Figure 1:**
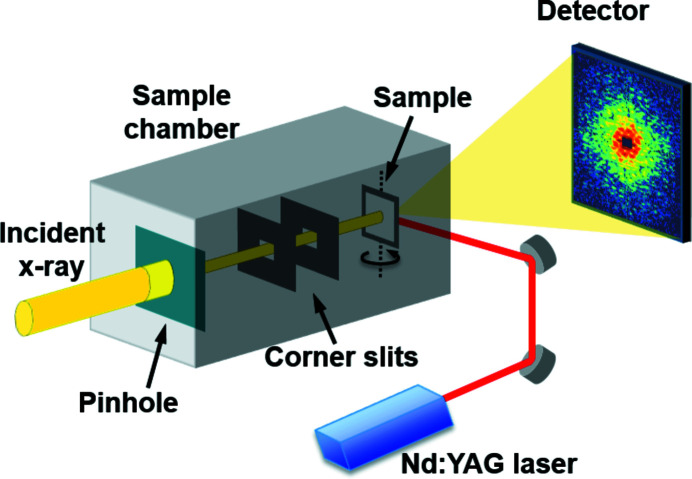
Schematic illustration of the CXDI setup. A beam-defining pinhole, corner slits, and sample are located inside a vacuum chamber and a nanosecond-pulsed Nd:YAG laser system with 532 nm wavelength is installed next to the sample chamber.

The CXDI experiment was performed at beamline 9C at Pohang Light Source-II (PLS-II) in Korea. A monochromated partially coherent 6 keV X-ray beam was focused by a toroidal mirror to increase the X-ray photon flux incident on a sample (Yu *et al.*, 2014 ▸; Kim *et al.*, 2017*a* ▸). A 10 µm-diameter tungsten (W) pinhole, fabricated by a focused ion beam process, was positioned near the focal plane of the toroidal mirror to achieve sufficient coherency at the sample plane which was located about 300 mm downstream of the beam-defining pinhole. The transverse coherence lengths at the sample plane were around 3.7 µm (H) × 4.3 µm (V) (Kim *et al.*, 2017*a* ▸). Two sets of slits were placed in front of the sample to eliminate parasitic scattering from upstream optics, especially from the beam-defining pinhole. A nano-second pulsed Nd:YAG laser system with 532 nm wavelength was installed near the sample chamber, with the 1064 nm wavelength component removed by using a dichroic mirror. The laser beam was guided through two right-angle (90°) mirrors and delivered to the sample inside the chamber through a view port. The efficiency of each optics component was above 90% and the distance between the laser source and the sample plane was about 1 m. The near-field laser beam was circular with a diameter of about 3.2 mm and the divergence of the laser beam was about 4.4 mrad. The laser beam power was about 0.2 W operating at 20 Hz repetition rate. The diffraction intensities of the AuNi alloy samples were measured using a single-photon-counting pixel detector with 981 × 1043 pixels of 172 µm pixel size (PILATUS 1M). The detector was located about 3.2 m downstream of the interaction point, and a beamstop was positioned in front of the detector to prevent damage from the intense transmitted direct X-ray beam. A schematic of the experimental setup is illustrated in Fig. 1 ▸.

A phase-separated AuNi alloy nanocrystal sample was fabricated by using the solid state dewetting process on a patterned Ni–Au bilayer film. We first patterned a 0.5 µm (H) × 2 µm (V) rectangle on a Si_3_N_4_ membrane window by e-beam lithography on which Ni and Au films, each of 10 nm thickness, were deposited by e-beam deposition. The pattern dimensions were selected by considering the relatively short X-ray coherent length in the horizontal direction (Kim *et al.*, 2017*a* ▸). The patterned Ni–Au bilayer film was then annealed thermally for 20 min at 850°C in vacuum, which resulted in a number of phase-separated AuNi alloy nanocrystals. The AuNi alloy nanocrystal specimern thus prepared was mounted at the position where the laser and X-ray beam cross each other inside a vacuum chamber.

## 3. Results and discussion   

### 3.1. Formation process of metastable mixed AuNi phase by laser annealing: two-dimensional projection CXDI   

We first performed a series of two-dimensional projection CXDI measurements to investigate the effect of laser irradiation on the AuNi nanoparticles. Coherent X-ray diffraction patterns of the AuNi sample in its initial as-prepared state and after exposing it to 5, 10, 50, and 250 laser pulses were recorded on the PILATUS detector. The phases of the diffracted waves, which were lost during the measurement process, were retrieved by hybrid input–output (HIO) (Fienup, 1982 ▸) and shrinkwrap (Marchesini *et al.*, 2003 ▸) algorithms and the quantitative real space images were obtained by the inverse Fourier transforms of the retrieved complex-valued diffracted waves. The image resolution was estimated to be better than 15 nm in the half-period resolution according to the phase retrieval transfer function (PRTF) (Chapman *et al.*, 2006 ▸) and the Fourier shell correlation (FSC) function (Vila-Comamala *et al.*, 2011 ▸) even although a pixel size of the reconstructed images is 15 nm (H) × 15 nm (V) (see Fig. S1 of the supporting information).

**Figure 2:**
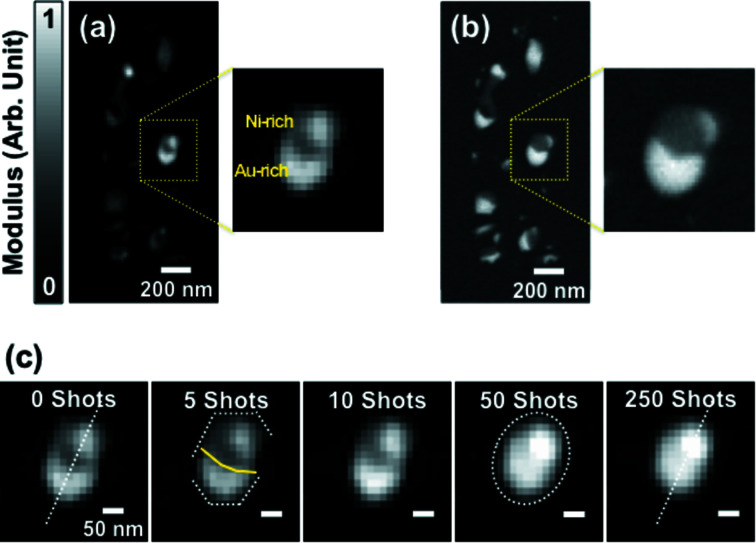
Reconstructed real space CXDI image (*a*) of the as-prepared AuNi sample and corresponding SEM image (*b*). The magnified images of the largest particle show phase-separated Au-rich and Ni-rich regions. (*c*) A series of reconstructed real space images of the largest particle after 0, 5, 10, 50, and 250 laser pulses irradiation.

The reconstructed real space image of the as-prepared AuNi sample is shown in Fig. 2(*a*) ▸, which is quite consistent with the corresponding scanning electron microscope (SEM) image shown in Fig. 2(*b*) ▸. We performed the SEM observation followed by CXDI measurement on the same particles. There exist a few AuNi nanoparticles formed as a result of the solid state dewetting process. The brighter parts represent regions of higher projected electron density along the beam direction. The magnified image of the largest particle shows that it consists of higher electron density and lower electron density regions, which correspond to Au-rich and Ni-rich regions, respectively. The AuNi particle was in the phase-separated state as predicted by the bulk phase-diagram. The SEM image shown in Fig. 2(*b*) ▸ also indicates that the AuNi particle was in the phase-separated state. The SEM image was obtained in the back-scattered electron (BSE) mode, which provides a better elemental sensitivity than the secondary electron (SE) mode. The dark Ni-rich area in the SEM image is larger than the corresponding area in the CXDI image. Considering that the SEM image is surface sensitive while the CXDI image represents a projected density profile, we conjecture that the Ni-rich region is segregated in the top part of the particle underneath which Au exists (Nguyen *et al.*, 1993 ▸; Lambrecht *et al.*, 1987 ▸; Cen *et al.*, 2017 ▸).

As the number of laser pulse irradiations increases, the Ni-rich and Au-rich regions mix with each other. The evolution of the AuNi particle during the laser annealing process is illustrated in Fig. 2(*c*) ▸. After exposing ten laser pulses to the sample, Au and Ni start to mix near the phase boundary, and the surface starts to become rounded, although the particle size in the major axis still remains the same as before. This indicates that the threshold number of laser pulses to induce the observed changes is about ten. The AuNi nanoparticle exhibited clear changes after exposed by 50 laser pulses including the rounding of the surface and mixing of Ni and Au regions. This indicates that the particle transforms to a non-equilibrium mixed metastable state (see also Fig. S2 of the supporting information).

**Figure 3:**
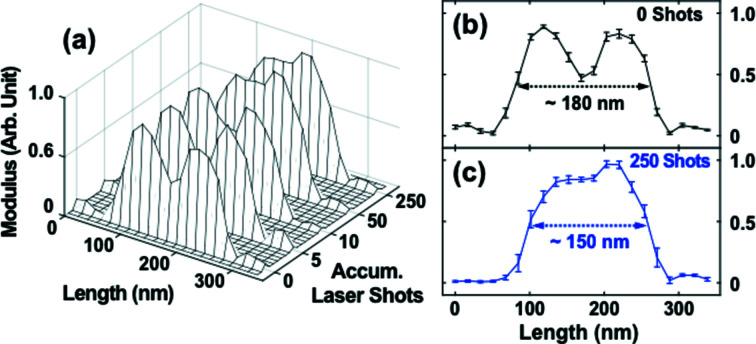
(*a*) Line profiles of the particles marked by white dotted lines in Fig. 2(*c*) ▸ (lines are not marked in the images of the 5, 10, and 50 laser shots). Line profiles before the laser irradiation (*b*) and after 250 pulses irradiation (*c*). Composition changes near the phase boundary and the rounding effect are clearly visible.

The line profiles of the AuNi particle along the white dotted line in Fig. 2(*c*) ▸ exhibit detailed behavior of mixing near the phase boundary. The peak positioned at 120 nm in the line profiles in the first three images (0, 5 and 10 accumulated laser shots) shown in Fig. 3 ▸ corresponds to the Au-rich phase, while the valley near 180 nm represents the Ni-rich region. The peak near 220 nm consists of the Ni-rich surface region, underneath which lies the Au-rich region that might have penetrated into the Si_3_N_4_ substrate (Nguyen *et al.*, 1993 ▸; Lambrecht *et al.*, 1987 ▸; Cen *et al.*, 2017 ▸). The line profiles of the last two images (50 and 250 accumulated laser shots) indicate that the phase boundary disappears and that the sample transforms to a non-equilibrium mixed state. There exists a small hump at 210 nm which represents the Au penetrated into the substrate. Figs. 3(*b*) and 3(*c*) ▸ illustrate the first and last line profiles shown in Fig. 3(*a*) ▸. Laser-induced formation of the non-equilibrium mixed state is clearly demonstrated. The dimension of the sample in this direction decreases, indicating the rounding in the shape.

We note that the mixing behavior is observed only in the largest particle with a size of over ∼200 nm among those shown in Fig. 2(*a*) ▸. No significant changes were observed in the smaller alloy particles, which is due to the efficiency of the laser energy absorption of those particles being much lower, which can be understood by Mie theory (Fales *et al.*, 2017 ▸; Metwally *et al.*, 2015 ▸). Laser absorption of Au is maximal when the particle size is around 200 nm. We think that the laser power used here was not sufficient to trigger changes in the smaller ones.

### 3.2. Three-dimensional visualization of the AuNi particles in the mixed state   

**Figure 4:**
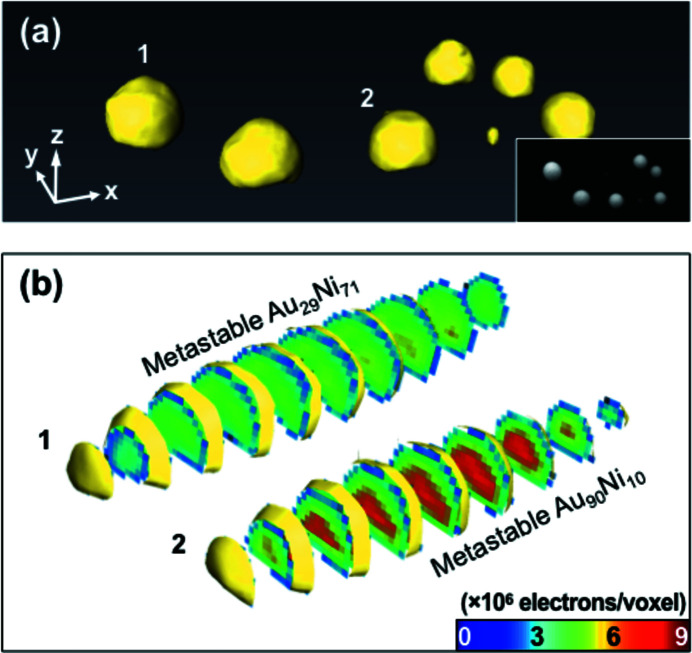
(*a*) 3D rendering of laser-annealed metastable AuNi nanoalloys. The inset shows an SEM image of the same sample. The length of the arrows is 100 nm. (*b*) A series of 2D sections of two individual particles, marked by #1 and #2 in (*a*), cut in the *y*–*z* plane with 23 nm intervals.

We performed a tomographic CXDI measurement on another AuNi nanoparticle sample in the mixed state in order to investigate the internal density profile in 3D non-destructively. The sample was prepared following exactly the same procedure as discussed previously with an irradiation by 20 laser pulses. The prepared sample was measured by SEM [see Fig. 4(*a*) ▸ inset] before the tomographic CXDI experiment. A set of 26 projected diffraction profiles, with an angular range from −69.44° to +63.43°, was measured by rotating the sample around a vertical axis perpendicular to the X-ray propagation direction following the equally sloped tomography (EST) (Miao *et al.*, 2005 ▸). Each 2D projection CXDI was reconstructed, firstly by using the guided hybrid input–output algorithm (GHIO) (Chen *et al.*, 2007 ▸), and then the EST algorithm was applied to reconstruct a 3D image. We measured 0° projection data sets at the beginning and at the end of the tomographic CXDI measurement to clarify any damage during the experiment. Both of the reconstructed real space images at 0° projection angle are compared and no significant differences are observed (see Fig. S3 of the supporting information).

The pixel size of the reconstructed 2D projection images for the tomographic CXDI study is 24.0 nm (H) × 11.3 nm (V) due to a rectangular Be window, in contrast to a wide Kapton window which was used for the two-dimensional projection CXDI experiment (Section 3.1), in front of the detector. As a result, the initial voxel size of the reconstructed 3D image was 24.0 nm (*y*) × 24.0 nm (*z*) × 11.3 nm (*x*) [the *x*, *y* and *z* axes are shown in Fig. 4(*a*) ▸]. Interpolation was applied to the *y* and *z* axes to make the voxels cubic, and the final voxel size was (11.3 nm)^3^. The incident X-ray flux was used to determine the electron density values of the reconstructed 3D image (Miao *et al.*, 2003 ▸; Kim *et al.*, 2017*b* ▸). In order to evaluate the incident X-ray flux, we monitored the current of a pin diode frequently and found it to be in the range 21.5 nA to 22.5 nA (22 nA ± 0.5 nA) when we measured the 0° data set. All the other angles are normalized to the total number of electrons of the 0° data set. Here, we expect about ±2% uncertainty of the incident X-ray flux. On top of that, the uncertainty of the sample position in the X-ray beam and reconstruction error may affect the electron density calculation.

Fig. 4(*a*) ▸ shows a bird’s eye view rendered image of the laser-annealed AuNi nanoparticle sample. The particles are relatively large compared with those shown in Fig. 2(*a*) ▸ and became spherical after laser annealing, indicating that their size is appropriate for the laser absorption. A series of cross sections of two selected particles, marked as #1 and #2 in Fig. 4(*a*) ▸, sliced at 23 nm intervals is shown in Fig. 4(*b*) ▸. The internal electron density of both particles is uniform indicating that they are not phase separated but in the mixed state. Each particle has a distinct average electron density. The electron density of particle #2 (6.4 × 10^6^ e voxel^−1^) is higher than that of particle #1 (4.7 × 10^6^ e voxel^−1^) which is slightly larger in size. The electron density of 100% Ni (Au) is 3.8 × 10^6^ e voxel^−1^ (6.8 × 10^6^ e voxel^−1^).

**Figure 5:**
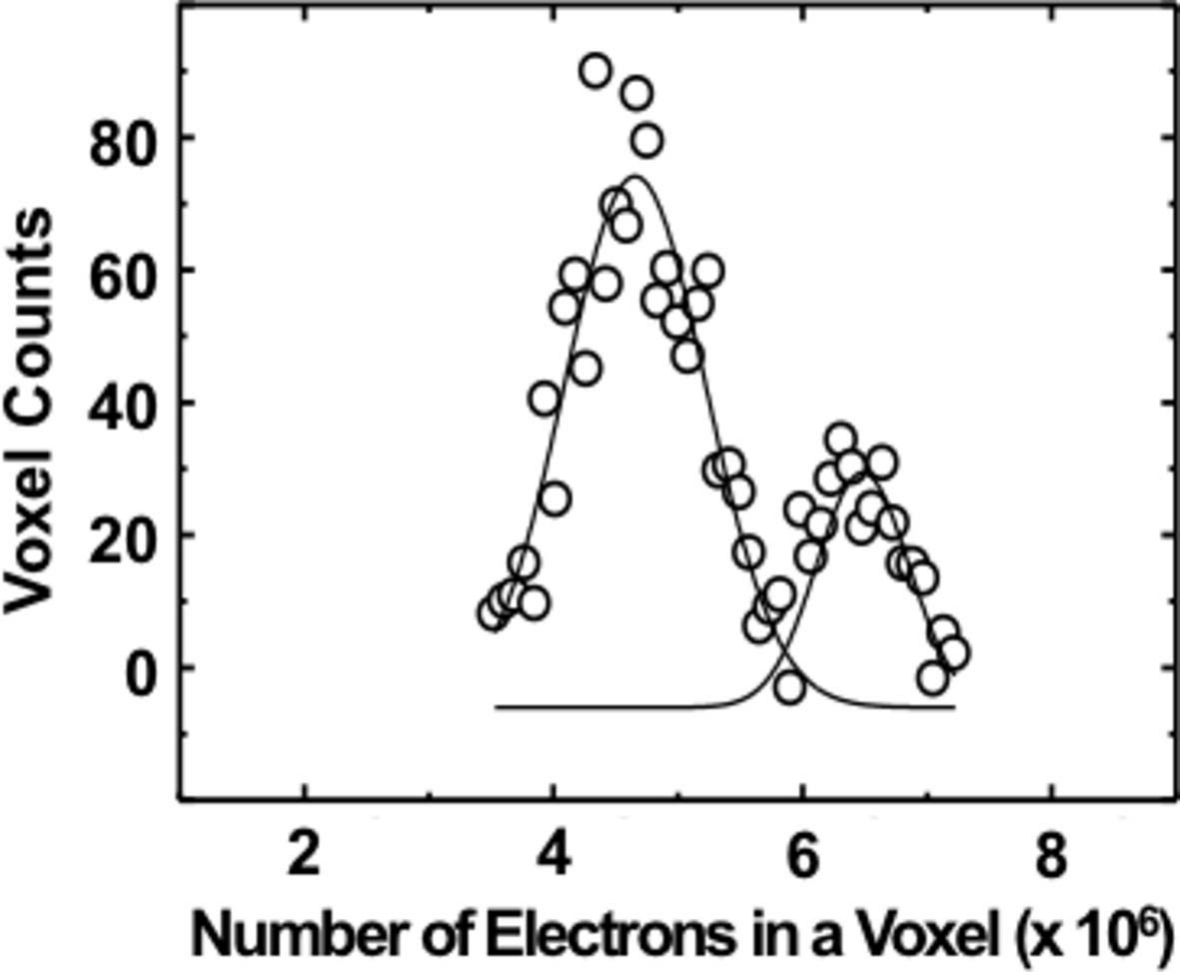
Histogram of the reconstructed 3D image (Gaussian fitting was applied after background subtraction). There are two major electron densities, 4.6 × 10^6^ and 6.5 × 10^6^, which correspond to 29 at% of Au and 90 at% of Au, respectively.

A histogram analysis of the electrons in voxels of the six largest particles shown in Fig. 4(*a*) ▸ indicates that there are two preferred compositions of the mixed phase. Fig. 5 ▸ shows a histogram where the number of voxels having electron numbers in a given interval is plotted. Two dominant electron densities, 4.7 × 10^6^ and 6.5 × 10^6^ e voxel^−1^, are found, which correspond to ∼29 at% (atomic %) and ∼90 at% of Au, respectively. However, the maximum electron density in the histogram is greater than the pure Au electron density which might be affected by the uncertainty of the incident X-ray flux and the sample position in the X-ray beam, the reconstruction error, and the interpolation of the reconstructed 3D image. Out of the six particles, three particles have a composition close to 29 at% of Au and the others have ∼90 at% of Au. It is interesting to note that the total number of electrons in all the particles is estimated to be ∼7.2 × 10^10^, which agrees well with the total amount of the deposited materials (2 µm × 0.5 µm × 10 nm Au and Ni) in the as-prepared sample, ∼7.3 × 10^10^ electrons.

## 4. Conclusion   

We have successfully visualized the laser-annealing processes of AuNi alloys by using the CXDI method. After a number of laser pulse irradiations, the phase boundary between Ni- and Au-rich regions fades away to form AuNi nanoalloy particles in a metastable mixed phase overcoming the miscibility gap. During the laser-annealing processes, the crystals become spherical. A three-dimensional CXDI shows that the internal part of the AuNi particles is indeed in the mixed phase. Two compositions, with ∼29 at% of Au and with ∼90 at% of Au, are preferred. We think that this study deepens the understanding of laser–matter interactions and provides a route to fabricate metastable metallic alloy nanosystems.

## Supplementary Material

Supporting information with three figures. Fig. S1: PRTF and FSC evaluations of the 2D projection CXDI experiment. Fig. S2: Line profiles of a series of the reconstructed real space images after 0, 5, 10, 50, and 250 laser pulses irradiation. Fig. S3: Reconstructed real space CXDI images measured at 0-degree projection angle in the beginning and in the end of the tomographic CXDI measurement for damage evaluation. DOI: 10.1107/S1600577520001617/yi5086sup1.pdf
