# Supplementary material for: Laser-induced metastable mixed phase of AuNi nanoparticles: a coherent X-ray diffraction imaging study
Source: J Synchrotron Radiat. 2020 Mar 31;27(Pt 3):725–9. doi: 10.1107/S1600577520001617 (PMC7206555; doi:10.1107/S1600577520001617)
Supplement: Supplementary file 1 [file s-27-00725-sup1.pdf]

Supporting information for article: Laser-induced Metastable Mixed Phase of AuNi nanoparticles: a Coherent X-ray Diffraction Imaging Study

Yoonhee Kim,[a,b] Chan Kim,[a,b]* Kangwoo Ahn,[b] Jungwon Choi,[b] Su Yong Lee,[c] Hyon Chol Kang[d] and Do Young Noh [b]*

[a]*European X-Ray Free-Electron Laser Facility, Holzkoppel 4, 22869 Schenefeld, Germany,* [b]*Department of Physics and Photon Science & School of Materials Science and Engineering, Gwangju Institute of Science and Technology, 61005 Gwangju, Korea,* [c]*Pohang Accelerator Laboratory, 37673 Pohang, Korea, and* [d]*Department of Materials Science and Engineering, Chosun University, 61452 Gwangju, Korea. E-mail: chan.kim@xfel.eu, dynoh@gist.ac.kr*

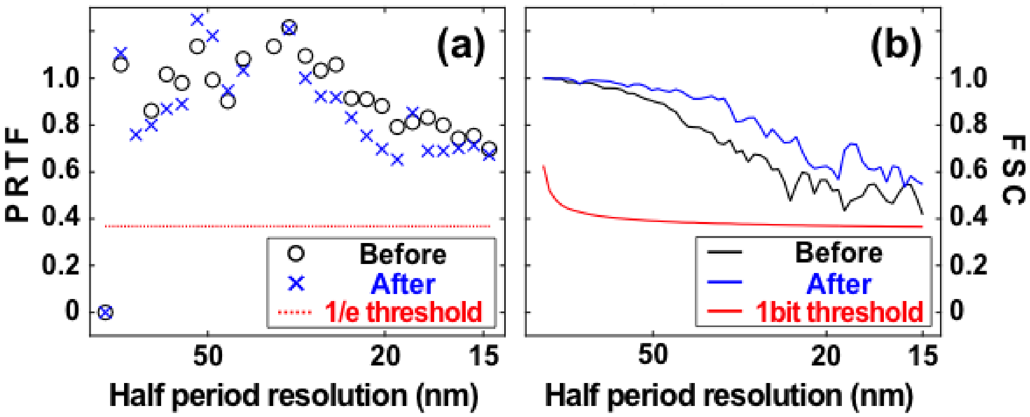

Fig. S1. (a) PRTF and (b) FSC evaluations before the laser irradiation (black circles & line) and after 250 pulses irradiation (blue crosses & line) of a series of two dimensional projection CXDI result. PRTF and FSC results were compared with 1/e and 1 bit threshold levels, respectively. In both cases, image resolution is better than 15 nm in half period resolution.

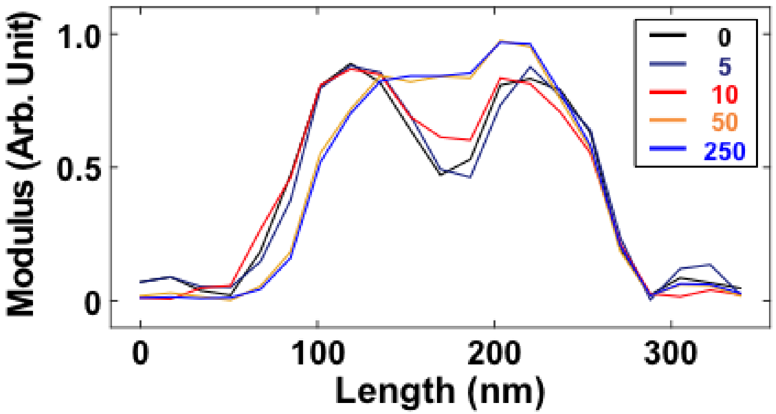

Fig. S2. Line profiles of the particles which are marked by white dotted lines in the main Fig. 2(c) (lines are not marked in images of 5, 10, and 50 laser shots). It shows exactly same line profiles in the main Fig. 3(a).

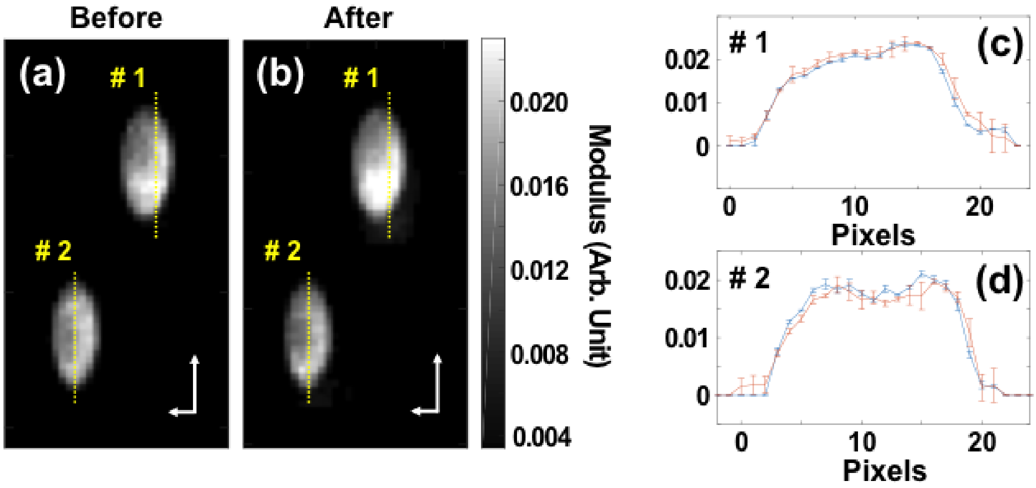

Fig. S3. (a, b) Reconstructed real space CXDI images measured at 0° projection angle (a) in the beginning and (b) in the end of the tomographic CXDI measurement for damage evaluation. (c, d) Line profiles of the particles which are marked by yellow dotted lines in (a) and (b). Blue and red lines correspond to before and after, respectively. No significant differences are observed.
